# Four-week “living high training low” program enhances 3000-m and 5000-m time trials by improving energy metabolism during submaximal exercise in athletes

**DOI:** 10.20463/jenb.2017.0060

**Published:** 2017-03-31

**Authors:** Hun-Young Park, Sungho Kim, Sang-Seok Nam

**Affiliations:** 1 Performance Activity and Performance Institute, Konkuk University, Seoul, Republic of Korea Republic of Korea; 2 Department of Sports Medicine, Kyung Hee University, Yongin-si, Republic of Korea Republic of Korea

**Keywords:** Living high training low (LHTL), Energy metabolism, Aerobic exercise performance, Time trial, Athletes

## Abstract

**[Purpose]:**

This study aimed to determine the effect of a 4-week living high training low (LHTL) versus a living low training low (LLTL) program on energy metabolism during submaximal exercise and 3000-m and 5000-m time trial (TT) in athletes.

**[Methods]:**

Male athletes (n = 20) were randomly assigned to the LLTL (n = 10, living at 1000 m and training at 700–1330 m) and LHTL (n = 10, living at simulated 3000 m and training at 700–1330 m) groups. We compared energy metabolisms during submaximal exercise on a treadmill and aerobic exercise performance (3000 m and 5000 m TT) before and after 4 weeks of training.

**[Results]:**

As expected, the LHTL group demonstrated enhanced energy metabolism during submaximal exercise via significant interaction (time × group) in heart rate, oxygen consumption, and carbon dioxide excretion; these variables were significantly decreased in the LHTL group compared with the LLTL group. Additionally, both training groups revealed significantly decreased blood lactate levels during submaximal exercise, 3000 m TT, and 5000 m TT but significant interactions (time × group) in the 3000 m and 5000 m TT. Thus, the LHTL group demonstrated greater improvements in 3000 m and 5000 m TT than the LLTL group via significant interactions.

**[Conclusion]:**

Our results suggest that 4-week LHTL intervention enhances 3000 m and 5000 m TT by improving energy metabolism during submaximal exercise. The proposed LHTL intervention in this study is a novel and effective method for improving aerobic exercise performance in male athletes.

## INTRODUCTION

Sports and exercise performance under normoxic conditions is highly related to metabolic and hemodynamic function, exercise economy, acid-base equilibrium capacity of the muscles, and oxygen delivering and utilizing capacity in the blood^22^. Training in natural or simulated altitude conditions is a common and popular practice for improving normoxic exercise performance via various hematological, physiological, biochemical, and structural changes that favor oxidative processes^7, 11^. 

However, several studies have examined the effect of training in natural or simulated altitude conditions on normoxic exercise performance^6, 17, 23^. These studies reported inconsistent results (positive or negative) due to differences in physiological characteristics, training conditions (exercise method, intensity, frequency, duration, and time), sports events, performance level, nutrition and medical support, fatigue level, and subject’s psychological state^16^. For these reasons, various natural or simulated altitude training regimens have been developed to enhance normoxic exercise performance. 

Training in natural or simulated altitude conditions can be divided into three regimens: (1) living high training high (LHTH) (residing and training under a natural or simulated altitude condition); (2) living low training high (LLTH) (residing at or near sea level but training under a natural or simulated altitude condition); and (3) living high training low (LHTL) (residing at a natural or simulated altitude condition but training at or near sea level)^16, 18^. Among these methods, LHTL intervention is widely recognized as the “gold standard” for normoxic exercise performance in athletes^2, 22^. 

The underlying mechanisms were generally increased hematological function via residing at natural or simulated altitude condition and metabolic function via training at or near sea level. These training effects lead to improved maximal oxygen consumption (VO_2max_) and oxygen flux rates during exercise^2, 3, 8^. The optimal “dose” of hypoxia is a very important issue for the successful LHTL intervention outcomes of athletic performance. Brugniaux et al^4^ and Park et al^16^ sought to determine the influence of factors such as the degree and duration of natural or simulated altitude condition on the balance between beneficial effects and potentially detrimental effects of LHTL intervention. They suggested that LHTL intervention should not exceed 3000 m for at least 18 days with a minimum of 12 hours per day of exposure. Therefore, using a randomized and controlled design,

this study aimed to investigate the effects of 4-week LHTL intervention with more than 16 hours at a 3000-m simulated altitude (14.5% O_2_) and more than 4 hours of training at 700–1330 m. We measured the effects on energy metabolism during submaximal exercise and exercise performance at sea level in athletes. 

## METHODS

### Participants

Our study included 20 male athletes (middle- and long-distance runners) who are registered in the Korea Association Athletic Federation. The subjects had not participated in any exercise and training program under a natural or simulated altitude condition in the previous 6 months; were non-smokers; and did not have any history of musculoskeletal, cardiovascular, or pulmonary disease. All athletes received information about the study purpose and process. They provided written consent after receiving a sufficient explanation of the experiment and understanding the possible adverse effects prior to the start of the study and were randomly assigned to the LLTL group (n = 10, living at 1000 m and training at 700–1330 m) and LHTL group (n = 10, living at a simulated 3000 m and training at 700–1330 m). All procedures were followed in accordance with the ethical standards of the responsible committee on human experimentation and the Helsinki Declaration. 

### Experimental design

Twenty male athletes in the LLTL (n = 10) and LHTL(n = 10) groups performed various training sessions dailyfor 4 weeks. The daily training sessions were performedfor >4 hours and consisted of dawn (warm-up, 90–100bpm; and 60 min of jogging, 130–160 bpm), morning(warm-up, 90–100 bpm; 150-m accelerated running fivetimes, 160–180 bpm; and 1000-m running six times,170–190 bpm), and afternoon exercise (warm-up, 90–100bpm; 300-m accelerated running five times, 165–190bpm; hill exercise, 160–180 bpm). Maximal heart rate(HR) was determined using the predicted HRmax formula(male = 206 – 0.69 × age)^12^. We designed a studyto verify the effectiveness of LHTL versus LLTL. Therefore,we analyzed energy metabolism during submaximalexercise on a treadmill with an absolute exercise intensityof 16 km/hr for 60 min and 3000 m and 5000 m TT wererecorded to evaluate exercise performance. 

The living in all groups occurred at the resort located at 1000 m in Taebaek city, Republic of Korea. The 3000-m altitude for the LHTL group was simulated using normobaric hypoxic environments by introducing nitrogen into the resort using a nitrogen generator (Separation & Filter Energy Technology Cooperation, Korea) with the capacity to simulate normobaric hypoxic environments for altitudes of up to 6000 m (9.7% O_2_). Patients in the LLTL group resided at 1000 m under comfortable conditions, similar to those in the LHTL group. The training in all groups was performed at 700–1330 m. The temperature within the resort was maintained at 24 ± 2^o^C and the humidity was maintained at 60 ± 5% for all conditions. 

### Measurements

#### Body composition

All participants fasted overnight prior to body composition measurements (i.e., height, weight, and body fat percentage). They wore lightweight clothing and were asked to remove any metal items. An X-SCAN PLUS (Jawon Medical, Korea) was used to measure height and body composition. 

#### Energy metabolism during submaximal exercise

Energy metabolism was evaluated using a Vmax-229 breath-by-breath auto metabolism analyzer (SensorMedics, USA), YSI-1500 lactate analyzer (YSI Inc., Yellow Springs, OH, USA), treadmill (Precor 932i, USA), and breathing valve in the facemask form. HR, minute ventilation (VE), oxygen consumption (VO_2_), carbon dioxide excretion (VCO_2_), and respiratory exchange ratio (RER) were measured before and after training while the athletes performed submaximal continuous exercises on a treadmill for 60 min at sea level and the summation values were used as the measurement values. Blood lactate levels were measured at 2, 4, 6, 8, 10, 20, 30, 40, 50, and 60 min of exercise, and the average values were used as measurement values. Blood (80 μL) was collected in a capillary tube using the fingertip method, and the sample was analyzed using a YSI-1500 lactate analyzer (YSI Inc.). All variables except for blood lactate level were measured every minute during the exercise. 

#### Exercise performance

To evaluate exercise performance, 3000 m and 5000 m TT were measured before and after training on an authorized 400-m athletic field at sea level in Suwon, Republic of Korea, at 9:00–10:00 AM (temperature 22–24°C, humidity 60–80%, and wind 0–10 km/h). 

### Statistical analysis

Statistical analyses were conducted using SPSS 23.0 for Windows (IBM Corp., Armonk, NY, USA). All data are presented as means ± SD. Two-way repeated analysis of variance (ANOVA) was used to identify the interaction and main effects between time and group. A post-hoc test within time and between groups was used with a paired and independent t-test. A priori, the level of significance was set at 0.05. 

## RESULTS

The subjects’ compliance and adherence to the study design was 100% in both groups based on the daily activity records. The characteristics of the selected athletes showed no significant intergroup difference (Table 1). 

**Table 1:** Participant characteristics

Variable	LLTL group	LHTL group
N	10	10
Natural or simulated altitude	Living 1000 m	Living 3000 m
	Training 700-1330 m	Training 700-1330 m
Age (yrs)	17.0 ± 1.3	17.6 ± 1.5
Height (cm)	173.1 ± 4.8	174.8 ± 7.4
Weight (kg)	55.8 ± 5.5	60.3 ± 6.9
Body fat (%)	11.9 ± 2.1	12.5 ± 1.5

### Energy metabolism during submaximal exercise

**Figure 1.**
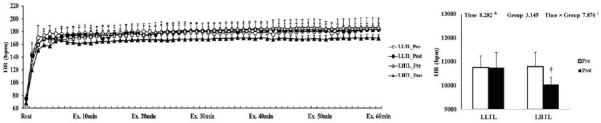
Changes in heart rate (HR) over 60 min (a) and summation (b) during submaximal exercise on a treadmill pre- and post-training in the control and intermittent hypoxic training groups. The bars indicate mean ± SD. *Significant interaction or main effect. †Significant difference between pre- and post-training. LLTL, living low training low; LHTL, living high training low.

**Figure 2.**
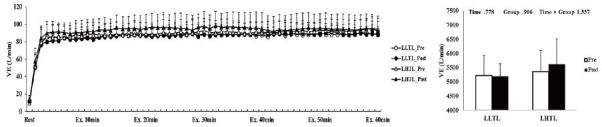
Changes in minute ventilation (VE) over 60 min (a) and summation (b) during submaximal exercise on a treadmill pre- and post-training in the control and intermittent hypoxic training groups. The bars indicate mean ± SD. LLTL, living low training low; LHTL, living high training low.

**Figure 3.**
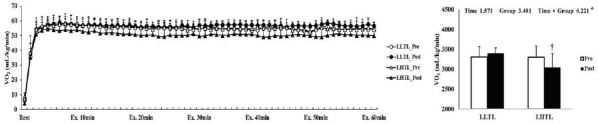
Changes in oxygen consumption (VO_2_) over 30 min (a) and summation (b) during submaximal exercise on a treadmill pre- and post-training in the control and intermittent hypoxic training groups. The bars indicate the mean ± SD. *Significant interaction or main effect. †Significant difference between pre- and post-training.

**Figure 4.**
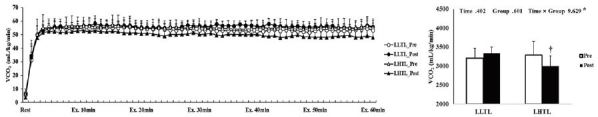
Changes in carbon dioxide excretion (VCO_2_) over 30 min (a) and summation (b) during submaximal exercise on a treadmill preversus post-training in the control and intermittent hypoxic training groups. The bars indicate mean ± SD. *Significant interaction or main effect. †Significant difference between pre- and post-training.

**Figure 5.**
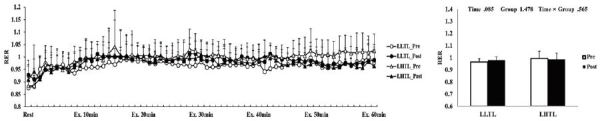
Changes in respiratory exchange ratio (RER) over 30 min (a) and summation (b) during submaximal exercise on a treadmill preversus post-training in the control and intermittent hypoxic training groups. The bars indicate mean ± SD.

**Figure 6.**
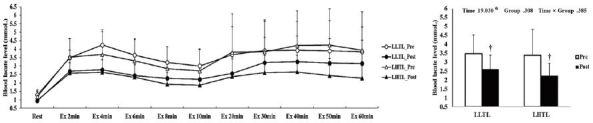
Changes in blood lactate level over 30 min (a) and summation (b) during submaximal exercise on a treadmill pre- versus post-training in the control and intermittent hypoxic training groups. The bars indicate mean ± SD. *Significant interaction or main effect. †Significant difference between pre- and post-training.

After the 4-week training period, there were significant interactions (time × group) in HR, VO_2_, and VCO_2_; these variables were significantly decreased by training in the LHTL group compared with the LLTL group (Figures 1, 3, and 4). In addition, blood lactate level showed a significant main effect within time; both training groups revealed a significant decrease and greater reduction tendency in the LHTL versus LLTL group (Figure 6). However, there were no significant differences in VE and RER (Figures 2, 5). 

### Exercise performance

As expected, there were significant interactions (time × group) in 3000 m and 5000 m TT; all groups presented a significant increase in 3000 m and 5000 m TT, but the LHTL group had higher improvement in all exercise performance variables compared with the LLTL group (Table 2). 

**Table 2:** Pre- versus post-training values of 3000 m and 5000 m time trials in the LLTL and LHTL group

Group	Time trial	Pre	Post	F-value	
LLTL	3000 m (second)	603.50 ± 26.42	584.00 ± 26.82†	Time Group Time × group	71.849* 1.764 8.611*
LHTL		599.00 ± 24.67	558.85 ± 26.71†		
LLTL	5000 m (second)	599.00 ± 24.67	558.85 ± 26.71†	Time Group Time × group	71.849* 1.764 8.611*
LHTL		1036.20 ± 38.68	997.30 ± 32.63†		

^*^ Significant interaction or main effect.

^†^ Significant pre- versus post-training difference.

## DISCUSSION

To our knowledge, the present study is the first to demonstrate that 4 weeks of LHTL intervention elicited increased exercise performance and improved energy metabolism responses during submaximal exercise in athletes. 

We found that the LHTL group had enhanced running economy due to decreased VO_2_ and HR during submaximal exercise on a treadmill for 60 min compared with the LLTL group. Running economy defined as the amount of energy spent per unit of distance is widely known as a determinant of exercise performance in athletes^21^. In particular, running economy is highly correlated with exercise performance^20^ and recognized as an accurate predictive factor of exercise performance^13^. The enhanced running economy is reportedly determined by three factors^10^: first, increased oxidative phosphorylation and carbohydrate use during submaximal exercise by the adaptation to natural or simulated altitude condition; second, an increased aerobic glycolysis process due to decreased VO_2_ and blood lactate levels; and third, increased oxygen utilization of mitochondria by adaptation to the natural or simulated altitude condition. A decrease in HR by LHTL intervention indicates enhanced running economy due to an increase in oxygen-delivering capacity, and it would be linked to decreased sympathetic nervous system activity and increased parasympathetic nervous system activity, resulting in α2-adrenergic receptor activation^15, 18^. In a previous study, Stray-Gunderson et al^24^ divided the athletes into three groups; LLTL, LHTH, and LHTL plus applied training. As a result, red blood cell counts and VO_2max_ were increased by the LHTH and LHTL intervention; however, the 5000-m TT was improved only in the LHTL group. This result was reportedly due to the LHTL group performing high-intensity interval training, which is essential for improving aerobic exercise performance at or near sea level, compared with the LHTH group. Liu et al^15^ applied 2-week LHTL intervention consisting of residing at a 1980-m simulated altitude for >12 hours and training at sea level. Decreased left ventricular end-systolic diameter and increased eject fraction, cardiac output, and stroke volume were observed, and these changes in the LHTL group suggest that the systolic function was enhanced by the increased left ventricle contractility, β-adrenergic receptor function, and energy utilization rate in the cardiac muscle. 

We also verified the effect of the 4-week LHTL intervention on enhanced respiratory metabolic response due to decreased VCO_2_ and blood lactate level during submaximal exercise on a treadmill for 60 min compared with the LLTL group. In general, enhanced aerobic energy metabolism by LHTL intervention decreased relative exercise intensity (i.e., HR) during exercise with the same absolute intensity after versus before training. Since relative exercise intensity decreases after training, VCO_2_ and blood lactate level, indicators of anaerobic metabolism, were reduced during the same absolute intensity exercise^25^. In addition, the increased exercise performance with LHTL intervention has been affected by peripheral hypotheses such as increased glycogen storage, fatty acid utilization, muscle buffering capacity, motor unit activation by stimulating the neuromuscular system, and changing muscle Na+-K+ ATPase activity and running economy^1, 3, 9, 20^. Here we considered that enhancing running economy through LHTL intervention improved VCO_2_ and blood lactate levels by increasing aerobic energy metabolic rate and consolidating tolerance and removal capacities of fatigue-causing substances during anaerobic energy metabolism^14, 17^. The improved energy metabolism response during submaximal exercise was possibly caused by sympathetic nervous system inhibition, parasympathetic nervous system hyperactivity, and chemical receptor adaptations in the central and peripheral nerves^5, 14^. 

Finally, based on the improved energy metabolism response during submaximal exercise, the main finding of our study was to observe an improvement in 3000 m and 5000 m TT after 4-week LHTL intervention compared with the LLTL group. These results are consistent with those of various previous studies^2, 4, 9, 19, 22^. However, when applying LHTL intervention to male athletes without erythropoiesis, the enhancement of 3000 m and 5000 m TT via improved energy metabolism (HR, VO_2_, VCO_2_, and blood lactate level) during submaximal exercise seems to be the original aspect of this study. 

Our results suggest that 4-week LHTL intervention effectively enhances 3000 m and 5000 m TT by improving energy metabolism (HR, VO_2_, VCO_2_, and blood lactate level) during submaximal exercise. The proposed LHTL intervention in this study can be considered a novel and effective method for improving aerobic exercise performance. Therefore, multiple facilities that can apply the LHTL intervention are needed to improve the competitiveness of Korean elite athletes. 
